# Gonadal Mosaicism as a Rare Inheritance Pattern in Recessive Genodermatoses: Report of Two Cases with Pseudoxanthoma Elasticum and Literature Review

**DOI:** 10.3390/cimb46090597

**Published:** 2024-09-11

**Authors:** Lisa Dangreau, Mohammad J. Hosen, Julie De Zaeytijd, Bart P. Leroy, Paul J. Coucke, Olivier M. Vanakker

**Affiliations:** 1 Center for Medical Genetics, Ghent University Hospital, 9000 Ghent, Belgium; lisa.dangreau@ugent.be (L.D.); paul.coucke@ugent.be (P.J.C.); 2 Department of Biomolecular Medicine, Ghent University, 9000 Ghent, Belgium; 3 Department of Genetic Engineering and Biotechnology, Shahjalal University of Science and Technology, Sylhet 3114, Bangladesh; jakir-gen@sust.edu; 4 Department of Ophthalmology, Ghent University Hospital, 9000 Ghent, Belgium; julie.dezaeytijd@ugent.be (J.D.Z.);; 5 Division of Ophthalmology, The Children’s Hospital of Philadelphia, Philadelphia, PA 19104, USA

**Keywords:** pseudoxanthoma elasticum, gonadal mosaicism, systematic literature review, genodermatosis, *ABCC6*

## Abstract

Germline mosaicism in autosomal recessive disorders is considered a rare disease mechanism with important consequences for diagnosis and patient counseling. In this report, we present two families with PXE in which paternal germline mosaicism for an *ABCC6* whole-gene deletion was observed. The first family further illustrates the clinical challenges in PXE, with a typical PXE retinopathy in an apparently heterozygous carrier parent. A systematic review of the literature on gonadal mosaicism in autosomal recessive genodermatoses revealed 16 additional patients. As in most reported families, segregation analysis data are not mentioned, and this may still be an underrepresentation. Though rare, the possibility of germline mosaicism emphasizes the need for variant verification in parents and sibs of a newly diagnosed proband, as it has significant implications for genetic counseling and management.

## 1. Introduction

Gonadal or germline mosaicism refers to the presence of two distinct cell populations in the gonads, one of which contains a genetic aberration. It is a well-known cause for autosomal dominant genodermatoses such as dystrophic epidermolysis bullosa, incontinentia pigmenti, neurofibromatosis type 1 and KID (Keratitis–Ichthyosis–Deafness) syndrome [1,2,3,4]. However, in autosomal recessive diseases, gonadal mosaicism seems rare. Among autosomal recessive genodermatoses, pseudoxanthoma elasticum (PXE; OMIM# 264800) is considered a paradigm disorder, in which the fragmentation of mineralized elastic fibers results in dermal, ocular and cardiovascular symptoms. Cutaneous lesions are typically seen in the flexural areas and include (plaques of) papular lesions and increased skin laxity with excessive skin folds. Ocular symptoms entail in fundo abnormalities such as peau d’orange, comet tails and angioid streaks. The latter may lead to subretinal neovascularization and hemorrhage with subsequent vision loss. Cardiovascular symptoms arise due to media calcifications in midsized arteries and include peripheral artery disease and stroke [5,6,7,8,9,10,11,12]. PXE is most commonly caused by biallelic pathogenic variants in the *ABCC6* gene (ATP-binding cassette sub-family C member 6; OMIM* 603234), though rare patients have been reported with bi-allelic *ENPP1* (ectonucleotide pyrophosphatase/phosphodiesterase 1; OMIM* 173335) or *CYP2U1* (Cytochrome P450, Family 2, subfamily U, polypeptide 1; OMIM* 610670) variants; digenic inheritance with *ABCC6/GGCX* pathogenic variants (gamma-glutamyl carboxylase; OMIM* 137167) has also been reported [7,13,14]. Whereas parents of PXE patients are usually obligate heterozygous carriers, we report on two recently encountered families where this was not the case and gonadal mosaicism in one parent was demonstrated. We systematically review the literature on gonadal mosaicism in recessive skin disorders and discuss the importance of this rare disease mechanism for autosomal recessive genodermatoses.

## 2. Materials and Methods

**Molecular analysis of the *ABCC6*, *ENPP1* and *GGCX* genes.** Genomic DNA was isolated from whole blood (QIAamp blood kit, Qiagen^®^, Hilden, Germany) and the coding regions of the *ABCC6*, *GGCX* and *ENPP1* genes were amplified using an established protocol. Primer sequences are listed in Supplementary Table S1. Direct sequencing was performed using an Applied Biosystems 3730xl Sequencer^®^, with an ABI PRISM BigDye Terminator Cycle Sequencing Kit (Applied Biosystems^®^, Foster City, CA, USA). Nucleotide numbers were derived from cDNA *ABCC6* sequences (GenBank accession no. NM_001171).

**MLPA (multiplex ligation-dependent probe amplification) analysis.** MLPA is a sensitive and specific molecular technique used for the simultaneous detection and quantification of multiple target sequences in a single reaction. MLPA employs two hybridization probes that bind adjacent to each other on the target DNA, followed by a ligation step that joins the probes only when they hybridize correctly, thus allowing the amplification of specific sequences. The amplified products are then analyzed via capillary electrophoresis to determine the presence and relative quantities of the target sequences [15]. MLPA analysis of the *ABCC6* gene was performed using the SALSA MLPA kit PO92-B3 (MRC-Holland, Amsterdam, The Netherlands) according to the manufacturer’s recommendations. This kit contains 23 probes corresponding to *ABCC6* exons 2, 4, 5, 7–15, 17, 18, 21–28 and 30 and control probes for quality control. The PO92-B3 kit lacks probes for ABCC6 exons 1, 3, 6, 16, 19, 20, 29 and 31. As *ABCC1* is in close proximity to *ABCC6* (6.5 kb telomeric), an *ABCC1* probe was also included. The construction of the kit precludes the generation of signals from the *ABCC6* pseudogenes. MLPA fragments were detected using an ABI3130XL or ABI3730XL capillary electrophoresis system (Applied Biosystems, Foster City, CA, USA). The genemapper software v6.0 (Applied Biosystems, Foster City, CA, USA) was used to calculate fragment size and concentration, whereas the quantification analysis was performed using Coffalyzer (MRC Holland, Amsterdam, The Netherlands). All samples were tested in duplicate.

**Evaluation of uniparental disomy of chromosome 16.** Microsatellite marker analysis was performed using 5 polymorphic markers spanning chromosome 16 (D16S521b-D16S291-D16S283-D16S3113 and D16S511).

**Paternity testing.** Paternity testing was performed using the PowerPlex 16 system (Promega, Madison, WI, USA) kit according to the manufacturer’s guidelines, based on the analysis of Short Tandem Repeats (STR), short tenderly repeated DAN sequences that involve a repetitive unit of 2 to 7 basepairs. The PowerPlex 16 System is a multiplex STR system that allows the co-amplification and three-color detection of sixteen loci (fifteen STR loci and Amelogenin to determine the sex): Penta E, D18S51, D21S11, TH01, D3S1358, FGA, TPOX, D8S1179, vWA, Amelogenin, Penta D, CSF1PO, D16S539, D7S820, D13S317 and D5S818. These STR are very polymorphic, making them ideal markers for paternity testing, allowing to achieve a probability of (non-)paternity > 99.99%. One primer for each of the Penta E, D18S51, D21S11, TH01 and D3S1358 loci is labeled with fluorescein (FL); one primer for each of the FGA, TPOX, D8S1179, vWA and Amelogenin loci is labeled with carboxy-tetramethylrhodamine (TMR); and one primer for each of the Penta D, CSF1PO, D16S539, D7S820, D13S317 and D5S818 loci is labeled with 6-carboxy-4′,5′-dichloro-2′,7′-dimethoxy-fluorescein (JOE). All sixteen loci are amplified simultaneously in a single tube and analyzed in a single injection or gel lane. The 15 autosomal STR loci were amplified in a GeneAmp PCR System 9700 thermocycler (Applied Biosystems, Foster City, CA, USA). The separation and detection of PCR products were carried out with an Applied Biosystems 3730xl Sequencer (Applied Biosystems, Foster City, CA, USA), and genotyping was performed by comparison with the allelic ladder included in the kit, using GeneScan 2.1 software (Applied Biosystems, Foster City, CA, USA). Statistical analysis, including the calculation of the paternity index and determination of the probability of paternity, was performed using the analytics described by Gjertson and Brenner (most recent version on www.dna-view.com) [16].

**Systematic literature review.** For the literature review, Pubmed was systematically searched until 22 May 2024 using the following key words: “recessive” and “de novo mutation” not “X-linked”. The inclusion criteria were as follows:

- All article types describing original research were included (no reviews);
- Neither the date of publication nor the journal played a role in the selection;
- Only articles describing humans were considered;
- Only articles written in English were considered;
- Only articles of which the full text was available were included in the analysis.

**Figure 1:**
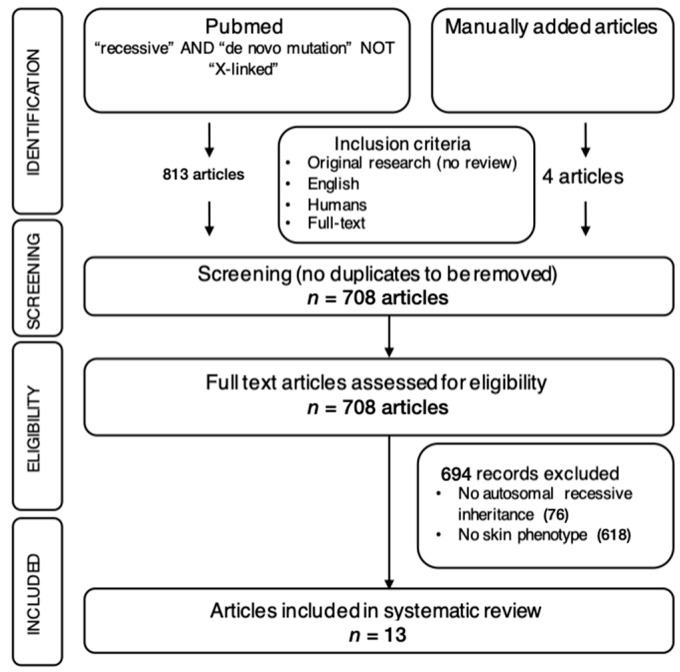
Systematic literature review approach represented as a PRISMA-P (Preferred Reporting Items for Systematic Reviews and Meta-Analysis Protocols) diagram.

The selection process followed the Preferred Reporting Items for Systematic Reviews and Meta-Analysis Protocols (PRISMA-P) and is shown in Figure 1.

## 3. Results

### 3.1. Case Reports

**Figure 2:**
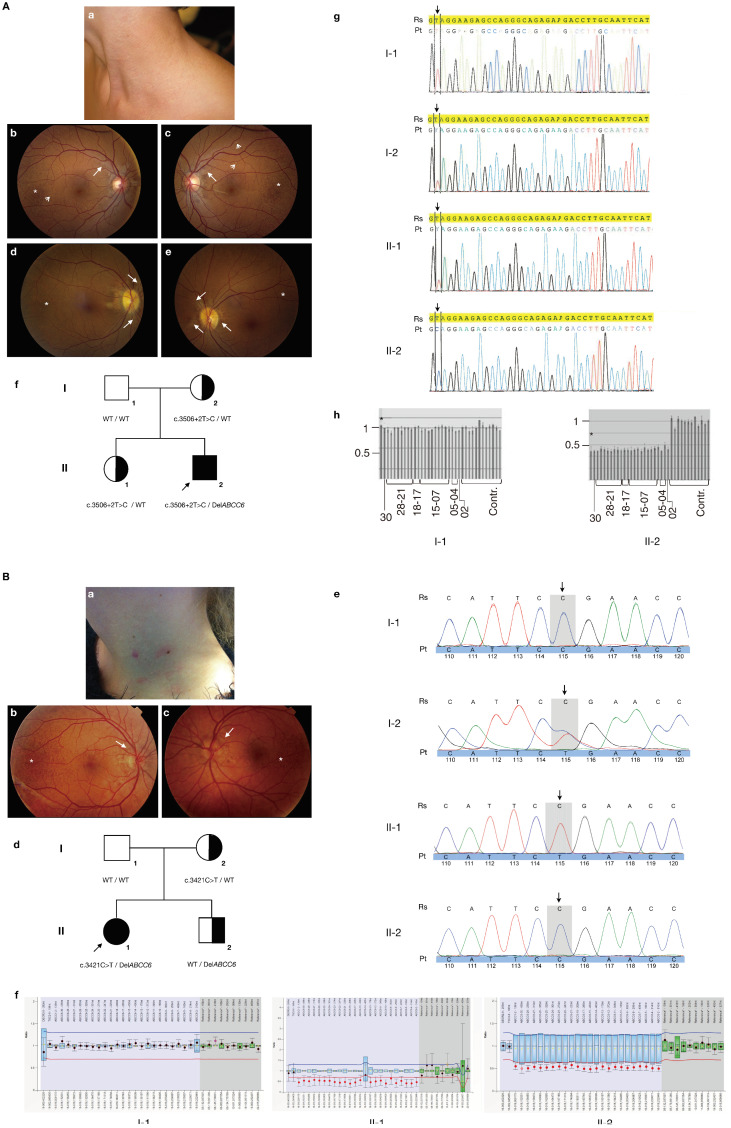
Clinical characteristics, pedigrees and molecular findings of the proband families. Panel (**A**): proband 1. (**a**) Papular skin lesions in the lateral neck at the time of diagnosis; (**b**–**e**) white-light funduscopy of the proband (**b**,**c**) and his mother (**d**,**e**). Peau d’orange (asterisk), comet tails (open arrows) and angioid streaks (closed arrows) are shown. (**f**) Pedigree of the family of proband 1 (II-2, arrowed). The *ABCC6* genotype is indicated for each individual (WT = wild type). (**g**) Electropherograms of the *ABCC6* direct sequencing results for individuals I-1, I-2, II-1 and II-2. The location of the affected nucleotide at position c.3506 + 2 is arrowed. Due to the presence of a heterozygous whole-gene deletion, only a single peak is observed in the proband, in contrast to overlapping peaks in his mother and sister. (**h**) MLPA results of the proband (II-2) and his father (I-1). Every bar is the ratio is the result of 1 probe pair PCR product. From left to right: ratio for the *ABCC1* probe, *ABCC6* exon 30, exon 28-21, exon 18-17, exon 15-7, exon 5, exon 4 and exon 2, followed by 12 bars representing the control probes (contr.). The *ABCC1* control probe is indicated with an asterisk. All ratios for the control samples are ~1, indicating that there is no deletion or duplication present. In the father (I-1), all ratios also equal to 1 indicate that no deletion is present; in the proband (II-1), all *ABCC6* probes and the ABCC1 probe have a ratio of ~0.5, confirming the presence of a heterozygous *ABCC6* whole-gene deletion. The deletion of *ABCC1* is commonly associated with *ABCC6* deletions and confirms that also exon 1 of *ABCC6*—for which no probe is present in the MLPA kit—is deleted; the *ABCC1* deletion however has no phenotypic consequences as was previously described [17]. Panel (**B**): proband 2. (**a**) Papular skin lesions in the lateral neck at the time of diagnosis; (**b**,**c**) white-light funduscopy of the proband. Peau d’orange (asterisk) and angioid streaks (closed arrows) are shown. (**d**) Pedigree of the family of proband 2 (II-1, arrowed). The *ABCC6* genotype is indicated for each individual (WT = wild type). (**e**) Electropherograms of the *ABCC6* direct sequencing results for individuals I-1, I-2, II-1 and II-2. The location of the affected nucleotide at position c.3421 is arrowed. (**f**) MLPA results of the proband (II-1), her father (I-1) and her brother (II-2). The ratio results of the probe pair PCR products are indicated by the dots; black dots: the ratio lies within the 95% confidence interval (CI) of the reference sample population, indicating the absence of a deletion or duplication; red: the ratio lies out of the 95% CI and over the arbitrary borders (0.7 to 1.3 by default), indicating the presence of a deletion or duplication. Whiskers: 95% CI for sample value (test or reference). Boxes: 95% CI in reference sample population (by default). Blue: compared to test probes; green: compared to reference probes. From left to right: ratio for the *TSC2* probe, *ABCC1* probe, *ABCC6* exon 30, exon 28-21, exon 18-17, exon 15-7, exon 5, exon 4 and exon 2, followed by 10 bars representing the control probes (reference*). All ratios for the negative control sample are ~1, indicating that there is no deletion or duplication. In the father (I-1) all ratios also equal to 1, indicating that no deletion is present; in the brother (II-2) and the proband (II-1), all *ABCC6* probes and the *ABCC1* probe again have a ratio of ~0.5, confirming the presence of a heterozygous *ABCC6* whole-gene deletion.

The first male proband presented at age 11 with typical papular PXE skin lesions in the neck as well as angioid streaks and peau d’orange in fundo (Figure 2A(a–c)). The diagnosis was histologically confirmed and further workup revealed abdominal and testicular calcifications; ultrasonography of the heart and vasculature was normal. In the years following the diagnosis, a progression of the extent of the skin lesions was noted. Physical examination of the proband’s parents and sister was normal except for a typical PXE retinopathy with angioid streaks, limited peau d’orange and comets in the mother (Figure 2A(d,e)). *ABCC6* analysis revealed compound heterozygosity for the pathogenic c.3506 + 2T > C splice site variant (via direct sequencing; Figure 2A(g)) and a whole *ABCC6* gene deletion (via MLPA; Figure 2A(h)) in the proband. Segregation analysis confirmed that the mother carried the c.3506 + 2T > C variant, while the second variant was not identified in a blood sample nor in buccal cells of the father (Figure 2A(f)). There was no indication for high-level mosaicism for the deletion in the proband, which would confirm that it was due to a postzygotic event. Unfortunately, a sperm sample of the father was not available due to a previous vasectomy; non-paternity (Supplementary Table S2) and maternal uniparental disomy (UPD) of chromosome 16 were excluded. The proband’s asymptomatic sister also carried the c.3506 + 2T > C variant (Figure 2A(f)). Given the ocular phenotype in the mother, analysis of the complete *ABCC6*, *ENPP1* and *GGCX* coding regions and MLPA testing of the *ABCC6* gene was performed but had a negative result.

The second proband was a female who was diagnosed at age 16 because of papular skin lesions in the lateral neck, which was confirmed using a skin biopsy (Figure 2B(a)). In fundo she had peau d’orange and a few small angioid streaks (Figure 2B(b,c)). Further work-up for cardiovascular symptoms or other sites of ectopic mineralization was normal and remains so to date at age 20. Physical examination of the proband’s parents and brother was normal. *ABCC6* analysis showed the proband to be compound heterozygous for the p.R1141* pathogenic variant (detected via direct sequencing), inherited from the mother, and a whole *ABCC6* deletion (demonstrated by MLPA), which was not found in the blood and buccal cells of either parent (Figure 2B(d–f)). High-level mosaicism in the proband, non-paternity (Supplementary Table S3) and UPD16 were excluded; the father refused for a sperm sample to be analyzed. Mutation analysis in the healthy brother also revealed, however, a heterozygous whole *ABCC6* deletion, strongly suggestive of paternal germline mosaicism (Figure 2B(d,f)).

### 3.2. Literature Review

Using a systematic literature review following the PRISMA-P guidelines, we identified 10 autosomal recessive genodermatoses where a suspected gonadal mosaicism was reported (Table 1). These include disorders with a predominant skin phenotype such as cutis laxa or epidermolysis bullosa as well as multi-systemic diseases such as Cockayne syndrome, Vici syndrome and PXE.

Almost all reports describe a single patient with suspected germline mosaicism, except for a report on epidermolysis bullosa with late-onset muscular dystrophy due to *PLEC1* pathogenic variants that reports two patients and the current report on PXE [18]. The phenotypes that were reported in the probands did not differ in symptoms or severity from other patients with the same disease. The parents with the suspected germline mosaicism did not present any symptoms as expected.

At the molecular level, nine of the pathogenic variants suspected to only be present in the germline of a parent were single-nucleotide variants, mostly nonsense variants. Though most of these variants were unique, a recurrent *COL7A1* variant, p.R1933*, was noted in dystrophic EB patients, which was suggested to be a hotspot variant as it occurred in a CpG dinucleotide [19,20,21]. The other variants were CNVs, mostly whole-gene deletions of *ERCC6, GORAB* and *ABCC6*, and one single exon duplication (in the *EPG5* gene) [21,22,23,24].

All but three cases were found to be due to paternal germline mosaicism. This assumption was usually based on the fact that one of the pathogenic variants of the proband was not found in the father and after paternity was confirmed. In only one of the reported families, genetic testing was performed on actual gonadal cells [19].

Specifically for PXE, after reviewing all previously reported PXE patients and families, only one family was found with similar segregation data. The proband was an 11 year-old boy who presented with typical papular skin lesions with middermal elastic fiber calcification and fragmentation on skin histology; ophthalmological symptoms were not yet present, which is not unexpected at this young age. He was compound heterozygote for p.R1164Q, which was inherited from the father, and had the p.R518* nonsense variant, which was not found in both parents and was suggested to be either a de novo mutation or reflecting germline mosaicism in the clinically unaffected mother. Similar to the two families in the current report, the clinical presentation or natural history was no different from other patients and families with PXE. There was no indication of a specific genotype–phenotype correlation [23].

**Table 1:** Results of the literature review: autosomal recessive genodermatoses with previously reported (suspected) germline mosaicism.

Disease	Gene	Variant Type	Parental Origin	N	Somatic Mosaicism	Semen Analysis	Paternity	Reference
Cockayne syndrome	*ERCC6*	Gene deletion	Maternal	1	NA	-	Yes	[21]
Cutis laxa	*GORAB*	Gene deletion	Paternal	1	NA	NA	Yes	[22]
Dystrophic EB	*COL7A1*	Nonsense	Paternal	1	NA	NA	Yes	[19]
	*COL7A1*	Nonsense	Paternal	1	No	NA	Yes	[20]
	*COL7A1*	Gene deletion	Maternal	1	NA	-	Yes	[25]
EB with late-onset muscular dystrophy	*PLEC1*	Nonsense	Paternal	2	NA	NA	Yes	[18]
	*PLEC1*	Nonsense	Paternal	1	NA	NA	Yes	[26]
Hermansky–Pudlak syndrome	*HSP1*	Nonsense	Paternal	1	NA	NA	Yes	[27]
Hypohydrotic ectodermal dysplasia	*EDAR*	Missense	Paternal	1	NA	NA	Yes	[28]
Junctional EB	*LAMB3*	Nonsense	Paternal	1	NA	Positive	Yes	[29]
Oculocutaneous albinism	*TYRP1*	Nonsense	Paternal	1	NA	NA	Yes	[30]
Pseudoxanthoma elasticum	*ABCC6*	Gene deletion	Paternal	2	No	NA	Yes	Current report
	*ABCC6*	Nonsense	Maternal	1	NA	-	-	[23]
Vici syndrome	*EPG5*	Exon 1 duplication	Paternal	1	NA	NA	Yes	[24]

EB: epidermolysis bullosa; N: number of patients; NA: not available.

## 4. Discussion

Finding an apparent ‘de novo’ pathogenic variant in a patient with an autosomal recessive genodermatosis remains rare. A systematic literature review was able to identify suspected gonadal mosaicism in 16 more patients from 14 families with a genodermatosis in which gonadal mosaicism was suspected (Table 1). Of these, only one patient had PXE [23].

Interestingly, in eleven families, the variants were due to paternal germline mosaicism. Indeed, it is known that most new pathogenic variants are observed in fathers and increasing paternal age positively correlates with the risk of new single-nucleotide variants [31]. In both our families the variant was however a copy number variant (CNV). Though many factors can contribute to the formation of CNVs, they usually occur due to a non-allelic homologous recombination between identical sequences of repeated DNA, which can occur during meiosis [32]. Indeed, several types of repeats (such as long and short interspersed nuclear elements and Alu repeats) are abundantly present in the intra- and extragenic region of *ABCC6,* making the gene more to for intragenic and whole-gene deletions [33,34]. It has been shown that these structural variations are more present on paternal chromosomes, emphasizing the contribution of the paternal germline to structural variation, though a link with paternal age has not always been consistent [35,36,37,38]. Indeed, in our report the first proband’s father was 42 years old at conception, while the second was only 30.

As in our cases, genetic testing is rarely performed on gonadal cells, except for one report [29]. Although a de novo occurrence can therefore not be formally excluded in the first family, recessive alleles are rarely attributable to de novo variants. Their number has been calculated to 1.1 × 10^–8^ per position per generation per haploid genome, and thus the likelihood that an individual is compound heterozygous and at least one of these variants arose de novo in this patient is very low [39]. The molecular result in the brother of the second proband confirms that paternal germline mosaicism must be present.

The reported cases of suspected germline mosaicism in recessive genodermatoses emphasize the importance of variant verification in the parents and sibs of a newly diagnosed patient to enable accurate preconceptional counseling. Even though they are difficult to estimate, in cases of germline mosaicism recurrence risks would be much lower than the traditional 25% recurrence risk for autosomal recessive disorders. Indeed, usually the recurrence risk for any apparent de novo pathogenic variant is considered 1%, though this may be an overestimation. Irrespective of the exact percentage, it implies that for a couple that has a first child with an autosomal recessive disease due to bi-allelic pathogenic variants, in which one of the partners is a heterozygous carrier and the other has (suspected) germline mosaicism, the recurrence risk of the same disease in future children would be low. This information may influence the choices couples make regarding whether or not to perform prenatal testing or pre-implantation genetic testing. In this regard, it should be noted that for a considerable number of probands, no segregation data were mentioned in the respective reports or case series. This may suggest that such events could occur more often than currently recognized. It also highlights the importance of profession genetic counseling to assure the proper assessment of all aspects of a genetic result. The possibility of germline mosaicism in autosomal recessive genodermatoses is also important to consider in preconceptional counseling in the context of expanded carrier screening, i.e., molecular carrier detection for a wide variety of recessive diseases based on incidence and disease severity, irrespective of family history. Some of these tests include autosomal recessive genodermatoses with observed germline mosaicism such as epidermolysis bullosa and PXE. If such carrier screening tests result in no shared pathogenic variants in overlapping genes in both partners, this may create a false sense of safety. This further stresses the need for careful genetic counseling by experienced professionals, taking into consideration in particular those disorders where germline mosaicism has been reported before.

The occurrence of gonadal mosaicism adds to the complexity of the clinical management of PXE, a very variable disease as demonstrated notably by the first family. As seen in the mother of proband 1, first-degree relatives of PXE patients may present phenotypic features of PXE. Heterozygous carriers of one *ABCC6* pathogenic variant have previously been shown to be more prone to cerebro- and cardiovascular diseases, while it was previously suggested that carriers in rare cases can also develop a PXE retinopathy [40,41,42]. Whether the retinopathy in the mother is a result of the identified heterozygous pathogenic variant however remains debatable and we consider it likely that pseudodominance is an explanation for the ophthalmological phenotype with the second variant in the mother being missed. Nonetheless, an ophthalmological evaluation of siblings and parents is appropriate upon the diagnosis of PXE in a proband. Though clinical management guidelines have been proposed for heterozygous carriers of an *ABCC6* pathogenic variant, these are obviously not applicable to obligate carriers with germline mosaicism [41].

## 5. Conclusions

We report two families demonstrating the presence of germline mosaicism in PXE. Though this remains rare, several other cases of recessive genodermatoses with parental germline mosaicism have been reported. The fact that in many case series and reports segregation analysis in parents is not mentioned may suggest that such an event could occur more often than is currently recognized. Such cases stress the need for the careful interpretation of molecular results, the importance to perform segregation analysis of identified pathogenic variants and the need of professional genetic counseling for diagnostic and expanded carrier screening.

## Data Availability

Data is contained within the article and Supplementary Materials.

## Supplementary Materials

The following supporting information can be downloaded at https://www.mdpi.com/article/10.3390/cimb46090597/s1: Table S1: Primer sequences for Sanger sequencing of *ABCC6*, *GGCX*, and *ENPP1*; Table S2: Paternity test results in family 1; Table S3: Paternity test results in family 2.
