# Supplementary material for: Gonadal Mosaicism as a Rare Inheritance Pattern in Recessive Genodermatoses: Report of Two Cases with Pseudoxanthoma Elasticum and Literature Review
Source: Curr Issues Mol Biol. 2024 Sep 11;46(9):9998–10007. doi: 10.3390/cimb46090597 (PMC11430005; doi:10.3390/cimb46090597)
Supplement: Supplementary file 1 [file cimb-46-00597-s001.zip › cimb-3126144-supplementary.pdf]

Supplementary Data file

Table S1. Primer sequences for Sanger sequencing of *ABCC6, GGCX* and *ENPP1*

Gene	Exon	Forward primer 5'-3'	Reversed primer 5'-3'
ABCC6	Exon 1	GGTCCAAAGTGTTTAGGAAGTC	GCAGCCCGAGAGATCTGCAGC
	Exon 2	GATCCAAAAAGTTGCCTGGC	TGTCCCCTGCCTCCCCCGAA
	Exon 3-4	TCCCAGTTGGACATGGGGC**C**	TATAAGTGTGTGCATCGTG**T**
	Exon 5	CCTCCTCTGTCTCCATTCCTTAT	AGACTGAGACCTCAAAGTGG
	Exon 6	CACAGTTCGTCCTGTCTTCCTAC	TGGCCCTGGAGAAGCAGCTGT
	Exon 7	GTGACTTACCCAGGGTCACAC	ATGATGAGCTTTTCTGAAG**T**
	Exon 8	CCCCCAACTCCCATGATTG**C**	AAGGATGCCACTAAGAGACC
	Exon 9	AGGCACCTCCTCTCACCAGC	GGTGACAGAGCAAGACTCC**A**
	Exon 10	TGGGTGGAGAAGCCACCTGG	GGGGGACTCCGTTCAAAT
	Exon 11	TCTGAGAGCTGGGCTCCTCG	CAGGACCTGTGGCTTCCTCC
	Exon 12	GGTGGCTGTCAGGGTGCAGG	CCACCTACCTCACCCTGCCC
	Exon 13	AGGCTGCCCTATCCATGCTTGC	GGAAGCTGGAGCCAGGTGTAG
	Exon 14	AGACACCGACACCCAAAACC	TGCTGGCTTGCCATTATGGG
	Exon 15	CTCTCCGATGCAGAGGCC	CTACACCACCTCTCAGGTG
	Exon 16	TCACAGAGGCGGGCTGAACC	AGTGGGACTTTCAGGATGGG
	Exon 17	AACCTATCCCACCTCTGTCC	TCGGGGACCCAAATGACTCC
	Exon 18	CAGTTTCACCCTGTAGATGC	ATGCTAAGTGCTTCCTCTGC
	Exon 19	CATGTGTGTAACTTCTACCC	GCCAGTAGGACCCTTCGAGC
	Exon 20	GTGTCAAAGTGGGTATCTGG	TCAGCTACTTCAGCTTCAGC
	Exon 21	CCTGAGGGTTAGGCACATAC	GCTACATTTGGTGGGAGGAC

	Exon 22	TCGCCTGGCCTTGCCTCAACC	GTAAAGGAGTCCTGAGCACC
	Exon 23	CCGCTCCTGAGGGTCTCCAG	GCTGGGTGAAACCTCATATATGG
	Exon 24	CTACCTCTCTATGTCTGTGG	ATATGACCTCAGGTCTCACC
	Exon 25	CTTGTGCCCAGAGAAGCATC	AGGGGTCCGACAGTCTCTG
	Exon 26	CTGTTGCAAGCCCTCAAGTG	AGCAGATGTCAACAGGGACC
	Exon 27	GTGAAGTCTTAGAGGAAGGC	TTGTCCCTGGAGTCCTTTGG
	Exon 28	AATGCCCACAAACCCTCTGG	ACCATGCCTCCCATCTTTGC
	Exon 29	AAAGATGGGAGGCATGGTG	TCAGAGCTTGGAATTGCAGATA
	Exon 30	ACCCACACATCACCATGTGC	ACTGCAGGGCTGCTGTGAGG
	Exon 31	AGAGCCTCTCTGTCTCCCTCT	TCCAGCACTGCAGGCTGTGC
GGCX	Exon 1	ACCGGGAGACACTGGCGTC	CTAGGGAAGCAAATTCTCCTG
	Exon 2	AGAGATTGTCATTCTCCACTCT	GAGCTGTTGGTGCAGTGATTTCT
	Exon 3	TTCACCAGCATGCTTCTATTTC	GGTTCTTGATGGTGCTAG
	Exon 4	ATGTCTTTCGCCGCAGGTAA	CCCCAGGGGAAAGTTACCAAG
	Exon 5	GGGTTGAAGGCTATTC C	CCCCTCAATGTTTACCT
	Exon 6	CATTACTGAGAGGATGCGTCACCT	TGTAACTCAGGAGCATGGATTC
	Exon 7	TGGCTAGTCCCTTCCTGCAAAACTG	TTCGTGCTGTGATGTGCTTTGA
	Exon 8	ATGTCTGATGCTGACAA	GAGCCCAGCCAAACTCCT
	Exon 9	CCCAGGGTTAAGGTAGCC	CTGGTTTTGCAGCCCCTTCTT
	Exon 10	AAGCAAGGGCTGTTCATCTTGG	TGTTGTGAACTTACCTGCGG
	Exon 11	AAGACAGAAAAGCCTCTCCTCA	GGTGGCCTGTGATGTCCTTAGAA
	Exon 12	TGCACTCAGTTCTTTCTGCTGTTG	GCCATGGGGTGGGATGATGAAC

	Exon 13	GGCTAGAACATCATTCATAACC	GGAGGCCATAAGCTGGCTAGAG
	Exon 14	TATGATGGCAATGACAAAATATTG	CTAGCTGGCAGAAGAGGAGTTC
	Exon 15	TGTCCATTGCATAGAATGGGTC	TTGTTGTGAACTTACCTGCGG
ENPP1	Exon 1	GCCCGAAATCAGACAGGAAG	GAAGAGGACGGTATGTCAAGG
	Exon 2	TCCAGAACAGGACATAAATCACAAG	CATAGGCACACATGCATCCAC
	Exon 3	GAGTTACACTCCACAGTGGT	TTCCGGGAGATCTTTTCCTG
	Exon 4	CCTGAAAATGGCTGCTGGAA	TGACATCACGTGTCCTTGGT
	Exon 5	ATCCAGAGGTGGAGGATGAG	GCCAAGATGCTTTCTTCCGT
	Exon 6	CAGTTGCATGCTGACCTCAA	AGTTCACCGCTTTGCAATGG
	Exon 7-8	TGCGATATGCCTAATAGCAG	CAGTTTGCCGAAATTCCTAC
	Exon 9	TCTCTGTCTCCTTCGAGGTT	CTCTGGATGGCCTTCCATTC
	Exon 10-11	AAAGTTCTGCGTCAGTTTCC	AACTGTAAGCAAAGCCAGTT
	Exon 12	CTCTGCCCTCTTTCCCTACA	TATGGCCTGGCCATATCCACAA
	Exon 13	GCAGATCTCTGTGAGCTCAAG	GCTACCCTCTCTTCACTCCA
	Exon 14	TCCTAGAGTTGCAGGTAGGAG	AGCACAGTAGGACATCATGAC
	Exon 15	CACTGTAACCCAGTGACACTC	TGAAGAACACCCTACACGTACCT
	Exon 16	CAGAGTAGGCATTCCAAGACC	ACTGTCGTTCTCTACACACTCA
	Exon 17	TTGACCACCTCCATGCACAA	CACATGAGTGCTGGTGACAAC
	Exon 18	CCCAGGTTCAGTTTCCAACA	TATACTCACCCAAGGGTCACA
	Exon 19	AAATTGCTGCTTCTCCTCTTCC	AGGCATACCTTTCACAAACCAG
	Exon 20	CATGAGTGCTCCGTTGTAGAG	AGGCAGATTTCTGAGTTTGAGG
	Exon 21	CATGACGATTGCCCCTAAC	ATTGGTGCTGACACTTGG

Exon 22	TGTTACTCTCCACCCCAAGA	GCACCAAAGAAGCACAAACC
Exon 23	ATACCATTCGCCAGTTCCAC	ATCTACACAGGGCTCACGA
Exon 24	CAGTGTTTCTCTGTGTAGAACAGG	GTTTCTTCCAGAGTGTGAGCAA
Exon 25	CGTGAACTGAGTGCTCTCTG	ATGAGCTACCCCAGTCCTT

Genbank accession no. for *ABCC6,* GGCX and ENPP1 are NM_001171, NM_000821.7, NM_006208.3 respectively. ABCC6-specific primers designed based on sequence differences between *ABCC6* and its two pseudogenes (*ABCC6-Ψ1* and *ABCC6-Ψ2*). Underlined nucleotides eliminate *ABCC6-Ψ2*, nucleotides in bold eliminate *ABCC6-Ψ1*.

Table S2. Paternity test results in family 1.

Family 1						
**Marker**	**Father (I-1)**		**Proband (II-2)**		**Mother (I-2)**	
D3S1358	15	16	15	**15**	**15**	17
TH01	9,3	9,3	**8**	9,3	7	**8**
D21S11	28	29	28	**29**	29	**29**
D18S51	13	14	14	**15**	**15**	17
Penta E	7	11	**5**	11	**5**	12
D5S818	12	13	**8**	12	**8**	12
D13S317	11	14	**11**	14	9	**11**
D7S820	10	10	**8**	10	**8**	12
D16S539	9	12	9	**11**	**11**	12
CSF1PO	11	12	11	12	11	12
Penta D	9	12	**2,2**	9	**2,2**	8
vWA	16	19	**15**	19	13	**15**
D8S1179	13	15	13	**13**	**13**	14
TPOX	8	8	8	**9**	**9**	11
FGA	21	24	**18,2**	24	**18,2**	20
Amelogenin	X	Y	**X**	Y	**X**	X

Allelic profiles are shown of the proband and his parents for the 15 STR loci and Amelogenin that were investigated. Results show that for each marker the proband inherited one allele from the father (underlined) and one allele from the mother (bold). In view of the frequency of the different fragments in the population, the probability of paternity is >99.999%.

Table S3. Paternity test results in family 2.

Family 2						
**Marker**	**Father (I-1)**		**Proband (II-1)**		**Mother (I-1)**	
D3S1358	15	17	15	17	15	17
TH01	7	19	7	**7**	**7**	7
D21S11	27	30	27	**30**	**30**	31
D18S51	16	18	16	**19**	18	**19**
Penta E	8	15	8	**15**	**15**	15
D5S818	11	13	**10**	13	**10**	12
D13S317	8	11	8	**8**	**8**	13
D7S820	10	10	10	10	10	10
D16S539	12	13	**12**	13	11	**12**
CSF1PO	8	11	11	**11**	10	**11**
Penta D	13	14	**10**	14	**10**	11
vWA	14	17	**16**	17	**16**	17
D8S1179	13	14	13	14	13	14
TPOX	8	8	8	**9**	8	**9**
FGA	23	27	**21**	23	**21**	23
Amelogenin	X	Y	X	**X**	**X**	X

Allelic profiles are shown of the proband and her parents for the 15 STR loci and Amelogenin that were investigated. Results show that for each marker the proband inherited one allele from the father (underlined) and one allele from the mother (bold). In view of the frequency of the different fragments in the population, the probability of paternity is >99.999%.
